# Aflibercept in wet AMD beyond the first year of treatment: recommendations by an expert roundtable panel

**DOI:** 10.1038/eye.2015.77

**Published:** 2015-07-09

**Authors:** M McKibbin, H Devonport, R Gale, M Gavin, A Lotery, S Mahmood, P J Patel, A Ross, S Sivaprasad, J Talks, G Walters

**Affiliations:** 1 Ophthalmology Department, St James's University Hospital, Leeds, UK; 2 Ophthalmology Department, Bradford Royal Infirmary, Bradford, UK; 3 Ophthalmology Department, The York Hospital, York, UK; 4 Ophthalmology Department, NHS Greater Glasgow and Clyde, UK; 5 Southampton General Hospital, Southampton, UK; 6 Manchester Royal Eye Hospital, Central Manchester University Hospitals NHS Foundation Trust, Manchester Academic Health Science Centre, Manchester, UK; 7 Centre for Ophthalmology & Vision Sciences, Institute of Human Development, University of Manchester, Manchester, UK; 8 NIHR Biomedical Research Centre at Moorfields Eye Hospital and UCL Institute of Ophthalmology, London, UK; 9 Bristol Eye Hospital, Bristol, UK; 10 Newcastle Eye Centre, Royal Victoria Infirmary, Newcastle upon Tyne, UK; 11 Department of Ophthalmology, Harrogate District Hospital, Harrogate, UK

## Abstract

This supplement has been sponsored by Bayer HealthCare. Please see acknowledgements for full disclaimer. Prescribing Information can be found in the appendices. L.GB.COM.05.2015.11280. Date of preparation: June 2015

This paper provides expert recommendations on administration of aflibercept in wet age-related macular degeneration (AMD) after Year 1 (Y1), based on a roundtable discussion held in London, UK in November 2014. The goals of treatment after Y1 are to maintain visual and anatomical gains whilst minimising treatment burden and using resources effectively. The treatment decision should be made at the seventh injection visit (assuming the label has been followed) in Y1, and three approaches are proposed: (a) eyes with active disease on imaging/examination but with stable visual acuity (VA) at the end of Y1 should continue with fixed 8-weekly dosing; (b) eyes with inactive disease on imaging/examination and stable VA should be managed using a ‘treat and extend' (T&E) regimen. T&E involves treating and then extending the interval until the next treatment, by 2-week intervals, to a maximum of 12 weeks, provided the disease remains inactive. If there is new evidence of disease activity, treatment is administered and the interval to the next treatment shortened; and (c) if there has been no disease activity for ≥3 consecutive visits, a trial of monitoring without treatment may be appropriate, initiated at the end of Y1 or at any time during Y2. Where possible, VA testing, OCT imaging and injection should be performed at the same visit. The second eye should be monitored to detect fellow eye involvement. In bilateral disease, the re-treatment interval should be driven by the better-seeing eye or, if the VA is similar, the eye with the more active disease.

## Introduction

Neovascular or wet age-related macular degeneration (AMD) accounts for 10–15% of all cases of AMD.^1^ In the UK alone it was estimated that there were 263 000 cases of wet AMD in 2007–2009, with an annual incidence of 39 700 new cases.^2^ The prevalence of wet AMD is rising, and is expected to increase by one-third by 2020.^2^ Without treatment, wet AMD leads to severe central vision loss.

Wet AMD is typically characterised by the growth of abnormal blood vessels from the choroid into the space under the retinal pigment epithelium or the neurosensory retina.^3^ These new vessels can leak serous fluid and/or blood, leading to a reduction in central vision.^3^ Clinical features of active wet AMD can include one or more of the following: sub-retinal fluid, intra-retinal fluid, sub-retinal haemorrhage, retinal pigment epithelial detachment, and intra-retinal exudates within the macula.^4^

Vascular endothelial growth factor (VEGF) plays a key role in intraocular neovascularisation in a number of conditions; it not only promotes angiogenesis (by stimulating vascular endothelial cell proliferation and migration) but also increases vascular permeability.^5^ VEGF-A, acting via the VEGF receptor 2, is thought to be the main stimulator of angiogenesis and vascular permeability in wet AMD.^5^

Anti-VEGF agents such as aflibercept (EYLEA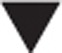; aflibercept solution for injection, Regeneron Pharmaceuticals, Inc, Tarrytown, NY and Bayer HealthCare Pharmaceuticals, Berlin, Germany) have become the mainstay of treatment for wet AMD. Aflibercept is a fusion protein, combining the key binding domains of VEGF receptors 1 and 2 and the Fc portion of immunoglobulin G. Dosing for aflibercept in wet AMD is initiated with one 2 mg injection per month for three consecutive doses followed by one 2 mg injection every 2 months during the first year. Monitoring between injections is not required. After 1 year of treatment, the treatment interval may be extended based on visual and anatomical outcomes (Table 1).^6^ In this event, the schedule of monitoring visits is determined by the treating physician and may be more frequent than the schedule of injections.

Aflibercept was licensed in Europe for the treatment of wet AMD in November 2012, and approved by the National Institute for Health and Care Excellence (NICE) in July 2013. Given the 90-day commissioning rule, all ophthalmology units in the UK would already be expected (as of November 2014) to have some patients who have completed 1 year of treatment with aflibercept. A national roundtable was therefore convened to discuss UK experience with aflibercept to date, and to use this experience, together with expert opinion, to develop recommendations on the practical application of aflibercept in wet AMD after Year 1. In particular, the discussion was based around maintaining the visual acuity (VA) gains from Year 1 and reducing treatment burden where possible. This paper will review the VEGF Trap-Eye: Investigation of Efficacy and Safety in Wet AMD (VIEW) study with aflibercept in wet AMD,^7, 8^ and the recommendations developed by the panel.

## Management of wet AMD in the UK

Guidance on the management of wet AMD in the UK has been published by NICE^9, 10^ and the Royal College of Ophthalmologists (RCO).^11^ It should be noted that the product label for ranibizumab has been changed since the RCO guidance was published, and therefore these guidelines are not presented below.

### NICE technology appraisals on ranibizumab and aflibercept in wet AMD

According to the NICE July 2013 technology appraisal (TA294), aflibercept is recommended as an option for treating wet AMD if it is used in accordance with the NICE recommendations for ranibizumab and offered at the discount agreed in the patient access scheme.^9^ The criteria for ranibizumab use (based on the NICE technology appraisal re-issued in May 2012; TA155) are as follows: best-corrected visual acuity (BCVA) between 6/12 and 6/96; no permanent damage to the central fovea; lesion size ≤12 disc areas in greatest linear dimension; and evidence of disease progression.^10^

### Treatment posology in the first year of wet AMD therapy

For ranibizumab, the label states that treatment should be given monthly until maximum VA is achieved and/or there are no signs of disease activity. Monitoring and treatment intervals can be extended thereafter.^12^ The treatment interval should be extended by no more than 2 weeks at a time in wet AMD.^12^

As outlined above, aflibercept treatment (2 mg) is initiated with one injection per month for 3 months. Thereafter, treatment continues at 2-monthly injections for the remainder of Year 1 (Table 1).^6^

## Aflibercept in clinical trials

### VIEW study design

VIEW 1 and 2 were two phase III, randomised, double-blind, multicentre, head-to-head non-inferiority trials that compared aflibercept with ranibizumab in patients aged ≥50 years with wet AMD.^7, 8^ VIEW 1 was conducted at 154 centres (*n*=1217) in the USA and Canada, while VIEW 2 was conducted at 172 centres (*n*=1240) in Europe (including the UK), the Middle East, Asia-Pacific and Latin America.^7^

Patients were randomised 1:1:1:1 to aflibercept 2 mg every 4 weeks (2q4), aflibercept 2 mg every 8 weeks (after three initial doses; 2q8), aflibercept 0.5 mg every 4 weeks (0.5q4) or ranibizumab 0.5 mg every 4 weeks (Rq4). Treatment continued to week 52.^7^ From weeks 52–96, patients were monitored each month and were treated either reactively or proactively in a ‘capped *pro re nata* (PRN)' protocol. ‘Reactive' treatment was based on the presence of disease activity (defined as: new or persistent fluid on OCT; increase in central retinal thickness of ≥100 *μ*m compared with lowest previous value; loss of ≥5 Early Treatment Diabetic Retinopathy Study (ETDRS) letters from best previous score in conjunction with recurrent fluid on OCT; new-onset classic neovascularisation; new or persistent leakage on fluorescein angiography; or new macular haemorrhage). ‘Proactive' treatment meant treating irrespective of disease activity. In VIEW, proactive treatment occurred if 12 weeks had elapsed since the last injection. Reactive treatment has been shown to result in worse VA outcomes than continuous therapy,^13, 14^ suggesting under-treatment in some patients, whilst it is generally accepted that proactive regimens can result in over-treatment in a proportion of patients.

### VIEW study results

Aflibercept results are shown for the 2q8 (licensed posology) arm only. Aflibercept was non-inferior (10% margin) to ranibizumab with respect to the primary endpoint, that is the proportion of patients maintaining vision (defined as losing <15 ETDRS letters) at week 52 (95.3% for aflibercept; 94.4% for Rq4). These proportions remained largely similar at week 96 (92.4 and 91.6%, respectively).^8^

**Figure 1:**
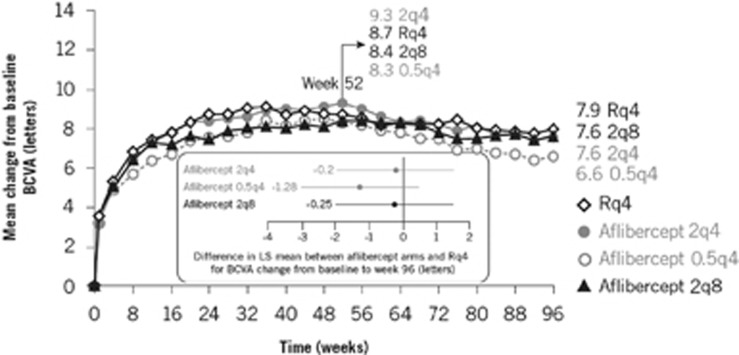
VIEW: mean change in best-corrected visual acuity (BCVA) from baseline to week 96.^8^ Reprinted from Ophthalmology, 121(1). Schmidt-Erfurth U, Kaiser PK, Korobelnik J-F, Brown DM, Chong V, Nguyen QD, *et al.* Intravitreal aflibercept injection for neovascular age-related macular degeneration: ninety-six-week results of the VIEW studies. p193–201 (2014), with permission from Elsevier. LS, least squares; q4, every 4 weeks; q8, every 8 weeks; R, ranibizumab.

**Figure 2:**
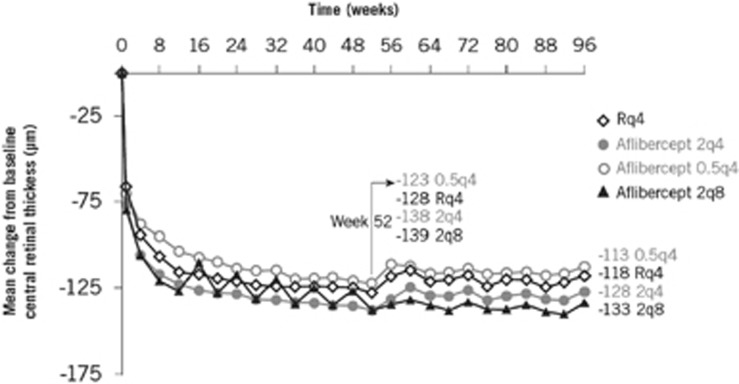
VIEW: change to central retinal thickness from baseline to 96 weeks.^8^ Reprinted from Ophthalmology, 121(1). Schmidt-Erfurth U, Kaiser PK, Korobelnik J-F, Brown DM, Chong V, Nguyen QD, *et al.* Intravitreal aflibercept injection for neovascular age-related macular degeneration: ninety-six-week results of the VIEW studies. p193–201 (2014), with permission from Elsevier. q4, every 4 weeks; q8, every 8 weeks; R, ranibizumab.

Mean BCVA increased and was sustained over 52 weeks with both aflibercept and ranibizumab. BCVA gains from baseline to weeks 52 and 96 were similar between aflibercept and ranibizumab (Figure 1). In general, aflibercept was similar to ranibizumab at weeks 52 and 96 for all other secondary efficacy endpoints examined, including the proportion of patients gaining ≥15 ETDRS letters, anatomical outcomes (change in choroidal neovascularisation [CNV] area, change in central retinal thickness [exploratory endpoint; Figure 2] and the proportion of eyes with a dry retina [*post-hoc* endpoint]) and change in National Eye Institute Visual Function Questionnaire-25 score.^7, 8^

Between weeks 52 and 96, the mean number of injections was 4.2 (standard deviation [SD], 1.7) and 4.7 (SD, 2.2) in the aflibercept and Rq4 groups, respectively. Overall, 15.9% of patients receiving aflibercept required frequent treatment (defined here as ≥6 injections between weeks 52 and 96) and 84.1% required less frequent treatment (<6 injections between weeks 52 and 96).^8^ Forty-eight per cent of patients in the aflibercept arm had three or fewer injections (ie the minimum number of injections, or fewer due to missed injections) between weeks 52 and 96.^15^

Both aflibercept and ranibizumab demonstrated a favourable safety profile. Serious ocular adverse events were infrequent, occurring in 3.9% of aflibercept-treated patients and 4.4% of ranibizumab-treated patients over the 96-week treatment period. Serious systemic adverse events were typical of those seen in an elderly population receiving intravitreal treatment for wet AMD. The incidence of Antiplatelet Trialists' Collaboration events was similar between aflibercept and ranibizumab.^8^

## National roundtable meeting

The roundtable meeting was sponsored by Bayer HealthCare. The expert panel was made up of 10 medical retina specialists and took place in London, UK on 5 November 2014. In advance of the meeting each participant was sent a questionnaire that was used to gather information about the number of wet AMD patients treated at their unit, the treatment distribution in their practice and the aflibercept treatment approach employed at each stage of management (up to 3 months, 3–11 months and 11 months onwards). Participants were also asked to provide audit data on the use of aflibercept in Year 1 at their institution, and to present these to the rest of the panel at the meeting. The questionnaire and audit data were used as the basis for the discussion of aflibercept use post-Year 1.

A summary of the recommendations generated by the expert panel is presented below.

## Recommendations for aflibercept after Year 1

**Figure 3:**
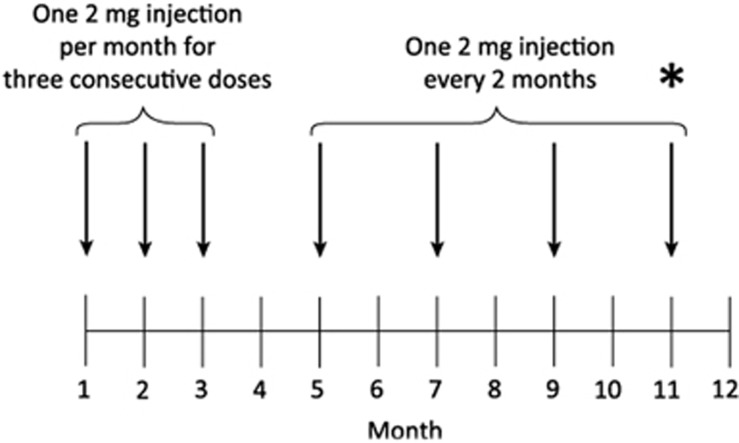
Dosing schedule for aflibercept in Year 1. *Indicates when the treatment approach for post-Year 1 is determined ie at the time of the seventh injection in Year 1.

It should be noted that, when referring to the label, the authors refer to month- rather than week-based dosing in this paper to reflect the licensed posology for aflibercept (stated in months). However, as most clinics are set up using weekly rather than monthly schedules, practice recommendations are given using week- rather than month-based dosing. As an example, ‘8-weekly' dosing is considered to be equivalent to ‘2-monthly' dosing. The dosing schedule for aflibercept in Year 1 is shown in Figure 3.

## Goals of treatment after Year 1

The goals of wet AMD treatment after Year 1 are to maintain visual and anatomical gains. These goals should be achieved by: individualising treatment regimens; minimising the treatment burden; and using resources cost-effectively. In the UK, this means delivering treatment within the local service framework and the NICE commissioning guidance. The recommendations presented here take into consideration the fact that clinic capacity limitations may have a bearing on treatment decisions; suggestions are provided for clinics where capacity is not an issue, and alternatives for clinics where capacity might be a limitation.

## Treatment approaches after Year 1

As yet, there is no way to predict the amount of treatment needed with either aflibercept or other anti-VEGF therapies for an individual. This is reflected in the large range of injection frequencies found in trials. For example, in the discontinuous arm of the Inhibition of VEGF in Age-related Choroidal Neovascularisation (IVAN) study, the 25% of eyes with the lowest injection frequency required <8 injections in total to the end of Year 2, and the 25% of eyes with the highest injection frequency required >17 injections in total (after three initial doses).^14^ Similar findings were observed in the Comparison of Age-related Macular Degeneration Treatments Trials (CATT). In Year 1, the mean number of injections for eyes receiving PRN treatment throughout Year 1 and Year 2 was 6.9 (SD: 3.0) for ranibizumab and 7.7 (SD: 3.5) for bevacizumab.^16^ The mean number of injections to the end of Year 2 was 12.6 (SD: 6.6) and 14.1 (SD: 7.0), respectively.^13^ This corresponds to approximately 5.7 injections in the ranibizumab arm and 6.4 injections in the bevacizumab arm in Year 2; the large SDs suggesting that many eyes required very little treatment. Likewise, in eyes switched to PRN treatment in Year 2, the mean number of injections was 5.0 for ranibizumab and 5.8 for bevacizumab in Year 2, but the SD values were again very wide (3.8 and 4.4, respectively).^13^

**Figure 4:**
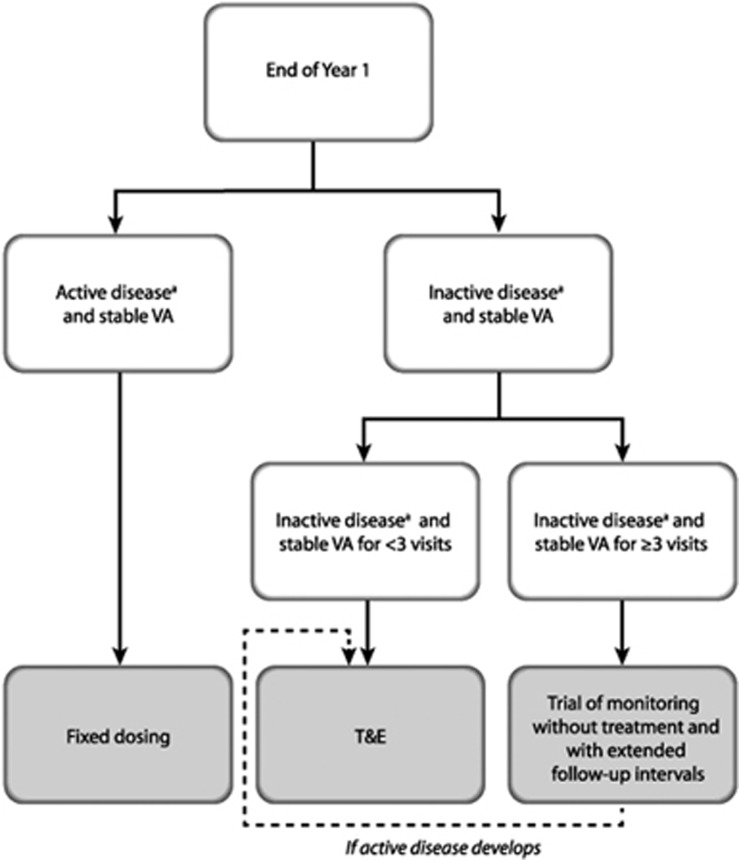
Algorithm for the treatment of wet age-related macular degeneration with aflibercept after Year 1. ^a^In the opinion of the treating physician. T&E, treat and extend; VA, visual acuity.

Recommendations for the use of aflibercept in wet AMD after Year 1 are presented below and in Table 2 and Figure 4. It should be noted that the expert panel did not feel that the VIEW study protocol for Year 2 (monthly monitoring with a combination of reactive and proactive therapy ie capped PRN) was a feasible option for many National Health Service (NHS) clinics owing to capacity constraints and patient expectation, and the VIEW study results suggest that this level of monitoring is not likely to be necessary for many patients.^8, 15^ This approach is therefore not suggested in the panel's recommendations. Three different treatment approaches are recommended for post-Year 1: (a) continuing with a fixed 8-weekly dosing regimen; (b) an individualised T&E regimen; and (c) a trial of monitoring without treatment and with extended follow-up intervals. The decision on the treatment approach after Year 1 should be made at the seventh injection in Year 1, based on the presence of active or inactive disease and taking into account the best VA and retinal anatomy achieved during the whole of Year 1. Whichever treatment approach is selected, OCT imaging should be performed and VA should be recorded at every visit post-Year 1 if possible; this should be undertaken in both eyes in order to detect fellow eye involvement early.

## Approach A: Fixed 8-weekly dosing

### Treatment strategy

This strategy involves continuing with proactive, fixed 8-weekly dosing. At the seventh injection visit in Year 1, the patient is re-injected and the next injection is scheduled for 8 weeks' time. OCT imaging and VA assessment are conducted at each visit if possible. Monitoring is not required between injections.

### Eyes suitable for fixed dosing

Eyes with active disease (in the opinion of the treating physician) but stable VA at the end of Year 1 should continue with fixed 8-weekly dosing after Year 1. The rationale for fixed dosing at this stage is, therefore, to continue regular treatment throughout the second year of treatment in eyes with persistent disease activity at the end of Year 1.

### Treatment burden and fixed dosing: progress to T&E after Year 2

Fixed dosing should continue throughout the second year of treatment. Thereafter, individualised T&E (see below) may be considered in order to minimise treatment burden and to maximise resource utilisation.

## Approach B: Individualised T&E

### Treatment strategy

The goal of T&E (an example of a proactive treatment regimen) is to find the optimal treatment interval at which patients can achieve maximum control of disease activity and stabilisation of VA with minimum treatment burden. T&E begins with an extension of the treatment interval after the seventh injection in Year 1. Treatment should be extended by 2-week intervals, up to a maximum of 12 weeks. The goal should be to maintain a ‘stable' outcome, defined by the treating physician in consultation with each patient and based on OCT imaging and VA examination. Both imaging and examination should be carried out at every visit if possible. Monitoring is not required between injections.

As an example, at the seventh injection visit in Year 1, the patient (who has completed an 8-weekly treatment interval) is reviewed and re-injected. If there is no evidence of disease activity on OCT imaging and examination (in the opinion of the treating physician) and the patient has stable VA, the treatment interval is extended by 2 weeks and the next injection is scheduled for 10 weeks' time. At this time, VA is measured, an OCT scan is performed and the patient is re-injected. If the patient has stable disease (in the opinion of the treating physician), the treatment interval is extended by a further 2 weeks, and the next injection is scheduled for 12 weeks' time. Once the patient has achieved a 12-week treatment interval, treatment should continue at this frequency throughout the second year of treatment.

The T&E regimen also allows the physician to shorten the treatment interval if there is new evidence of disease activity (in the opinion of the treating physician) and/or a reduction in VA. In these cases, OCT imaging is performed, VA is assessed and the patient is injected. The interval to the next treatment is shortened as appropriate. For eyes with a small increase in disease activity (e.g. those with some fluid upon OCT but stable VA), shortening the treatment interval by 2 weeks is recommended. Eyes with significant disease recurrence (e.g. those with new CNV activity, a symptomatic reduction in VA, extensive sub-retinal haemorrhage or a marked change upon OCT) should undergo more frequent monitoring, with treatment where necessary.

### Eyes suitable for T&E

At the end of Year 1, eyes with inactive disease (in the opinion of the treating physician) and stable VA are eligible for individualised T&E. The rationale for T&E, therefore, is to reduce the treatment burden and avoid unnecessary treatment in patients who have successfully achieved inactive disease at the end of Year 1.

### Treatment burden and T&E: use of virtual clinics

The T&E regimen is suitable for both a one-stop service (where OCT/VA and injection are performed on the same day) and a two-stop service (where OCT/VA and injection are performed on different days). In two-stop clinics, the use of ‘virtual clinics' can be employed to refine the patient pathway and improve capacity. Firstly, the injection visit is scheduled based on the OCT imaging and VA assessment from the previous visit. At the injection visit, OCT imaging is performed and VA is recorded prior to the injection. The OCT assessment then takes place either at the time the patient visits, or remotely (ie in the virtual clinic) at a later date by the consultant ophthalmologist or by a non-medical member of staff under supervision. The outcome of the OCT assessment determines when the patient is asked to come back for their next injection.

## Approach C: Trial of monitoring without treatment

### Treatment strategy

A trial of monitoring without treatment involves performing OCT imaging and VA assessment without giving an injection. The next planned monitoring visit should take place after an interval of between 4 and 8 weeks initially, increasing by 2-week increments, up to a maximum of 12 weeks.

If active disease (in the opinion of the treating physician) develops during a trial of monitoring without treatment, the patient should go back to T&E, starting at 8 weeks and extending out to 10 and 12 weeks, once stable disease is achieved once more.

### Eyes suitable for a trial of monitoring without treatment

At the end of Year 1 or at any time-point during Year 2, eyes that have had inactive disease and stable VA for at least three consecutive visits (including the current visit) may be considered for a trial of monitoring without treatment. A trial of monitoring without treatment avoids unnecessary treatment in patients who no longer require treatment, eliminating the treatment burden altogether.

The observation that almost half of all patients in the 2q8 arm of VIEW had three or fewer injections between weeks 52 and 96^15^ and data from the discontinuous treatment arms of IVAN and CATT^13, 16^ reinforces the fact that a trial of monitoring without treatment may be suitable for a substantial proportion of eyes. It is unknown whether the 12-weekly injections in VIEW Year 2 were proactive (ie protocol-mandated injections because 12 weeks had elapsed since the last injection), reactive (ie injection prompted by the presence of active disease) or a combination or proactive and reactive. As a result, it is unknown exactly how little or how much treatment these patients really needed.

### Treatment burden and a trial of monitoring without treatment: use of virtual clinics

Where necessary, a trial of monitoring without treatment can be conducted purely in the ‘virtual clinic' to improve capacity. As outlined above, OCT imaging and VA assessments can be conducted in the virtual clinic, while the OCT interpretation can be done online by the consultant or by non-medical staff, under supervision.

## Re-intensification of treatment

The panel discussed the options for eyes showing a greater treatment response during the initial monthly treatment phase than during the 2-monthly treatment phase. The interval between two doses of aflibercept should not normally be shorter than 8 weeks, however the RCO guidelines for wet AMD advise that hyperactive lesions may for a short time require more intensive therapy, at the discretion of the treating physician.^11^

Eyes with significant disease reactivation during treatment in Year 2 (for example those with a significant increase in fluid and a symptomatic drop in VA) or eyes with late disease recurrence after completing initial aflibercept treatment should receive more frequent monitoring, with treatment as necessary.

Given the complexity behind defining late disease recurrence after stopping treatment, however, further recommendations on managing late disease recurrence are beyond the scope of this article.

## Discharge strategy

Given that aflibercept has only been licensed in the UK for 2 years, very few patients have been discharged to date. As a result, the proposed discharge strategy is based on experience with only a small number of patients.

Patients with stable VA who have not received any treatment for a period of 12 months or more may be considered for discharge from an active treatment service either into virtual or community clinics, or without planned further review. These patients should be seen by an ophthalmologist in person to allow for a fully informed discussion. Discharge by letter, without any direct patient contact, under these circumstances is discouraged. One should bear in mind that, in most cases, discharge will only be an option beyond the second year of treatment, that is, beyond the scope of our recommendations.

Data from the SEVEN-UP study suggest that wet AMD is a chronic disease requiring chronic treatment.^17^ Consequently, patients with wet AMD may re-present some time after their last injection. Follow-up at regular intervals (at least once a year), for example with a community optometrist or in virtual clinics, can be considered as alternatives to discharge, in order to check for changes in visual function in either eye. If late disease recurrence develops during this time, the patient should return to the clinic for treatment. Treatment should not be re-initiated if the patient no longer meets the criteria outlined in the NICE TA for ranibizumab.^10^

## Fellow eye involvement

Once a patient has been diagnosed as having wet AMD in one eye, the risk of CNV in the fellow eye is high; in a study in patients with extrafoveal CNV, the 5-year incidence of CNV was reported to be 26%.^18^ Thus, it is important to monitor both eyes using OCT, to ensure that fellow eye involvement is detected early. There is increasing evidence to suggest that starting treatment with vision better than 6/12 maintains good vision,^19^ however it should be noted that this is outside of NICE guidance and may require an individualised funding request to local commissioners.

If a patient is having bilateral therapy, treatment intervals should be tailored to patient visits in order to synchronise treatment of both eyes. The better-seeing eye should drive the re-treatment interval for the worse-seeing eye. However, if visual function is similar in the two eyes (difference in VA between eyes ≤5 letters), then the eye with more active disease should drive the re-treatment interval. Patients should receive the same anti-VEGF therapy in both eyes.

## Other considerations

In terms of safety considerations, the risk–benefit profile should be discussed with the patient before initiating treatment and each time the treatment regimen is altered. It should be highlighted to the patient that there is a small, added risk of ocular adverse events associated with treatment, mostly related to the injection procedure.

An informed discussion with the patient is necessary to determine their treatment priorities or preferences. For example, some patients may be more willing to accept more frequent injections in order to try to achieve additional improvements in vision. Such a patient might be one who is keen to retain adequate visual acuity to continue driving. On the other hand, some patients may be unwilling to have very frequent injections and may be willing to accept some degree of disease activity. Examples include patients who have concerns about the risks associated with treatment, those who have experienced pain or an adverse event during or after injection, or those who have difficulty with regularly attending the clinic. The benefit to treatment burden may also vary according to whether the first or second eye is being treated.

## Revised re-treatment criteria

Recommended re-treatment criteria were developed by the panel at the roundtable meeting. The criteria were based on those used in Year 2 of the VIEW study, updated to reflect clinical experience. The definition of a ‘dry' or ‘wet' retina is highly variable between physicians, and therefore the revised criteria suggest that patients are re-treated when the treating physician considers there to be disease activity.

The recommended re-treatment criteria are as follows:

*Patients should be re-treated if, in the opinion of the treating physician, there is new or persistent disease activity, as indicated by one or more of the following (this list provides examples but is not exhaustive):*

- *New or persistent fluid as indicated by OCT, or increase in central retinal thickness compared with the lowest previous value as measured by OCT, or*
- *Loss of vision from the best previous VA if, in the opinion of the treating physician, this is because of disease activity, or*
- *New choroidal neovascularisation or new or persistent leakage on fluorescein angiography, or*
- *New macular haemorrhage*

## Conclusions

It is generally accepted that the VIEW study Year 2 protocol of monthly monitoring with capped PRN treatment is not feasible for many NHS ophthalmology clinics because of capacity constraints and patient expectation, and is not mandated by the aflibercept label. The recommendations given here offer alternative treatment pathways after Year 1; the intention is to maintain visual and anatomical gains whilst minimising the treatment burden and using resources cost-effectively. Three main treatment approaches are suggested: (a) continuing with a fixed 8-weekly dosing regimen for eyes with active disease and stable VA at the end of Year 1; (b) an individualised T&E regimen for eyes with inactive disease and stable VA at the end of Year 1; and (c) a trial of monitoring without treatment for eyes with inactive disease and stable VA for three or more consecutive visits either at the end of Year 1 or at any point in Year 2.

The rationale for fixed dosing at this stage is to continue regular treatment in eyes with persistent disease activity at the end of Year 1. Individualised T&E and a trial of monitoring without treatment, on the other hand, help to reduce the treatment burden and avoid unnecessary treatment in patients who have successfully achieved inactive disease by the end of Year 1. The use of virtual clinics, where the consultant ophthalmologist or a non-medical member of staff reviews OCT images remotely, can be a means of improving clinic capacity during T&E or a trial of monitoring without treatment.

Whichever treatment approach is selected, an informed discussion with the patient is necessary to determine their treatment priorities and their preferences regarding disease control and treatment frequency, bearing in mind the VA gains and the response to therapy during the first year of treatment.

## Figures and Tables

**Table 1:**
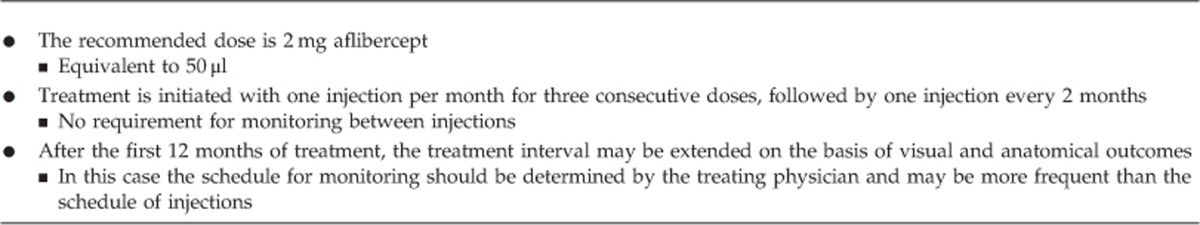
European posology of aflibercept in wet age-related macular degeneration^6^

**Table 2:**
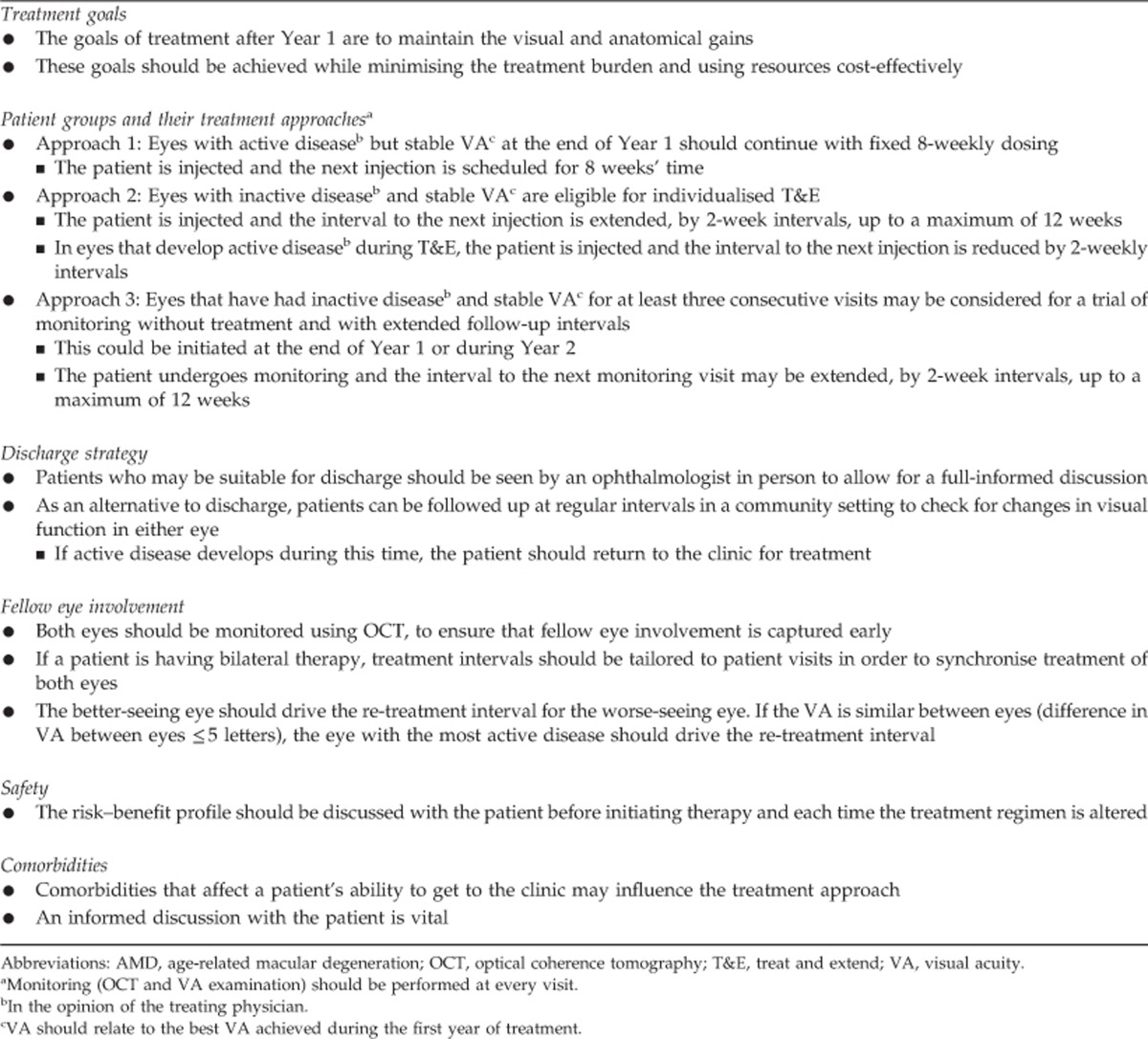
Summary of recommendations for aflibercept in wet age-related macular degeneration with aflibercept after Year 1

## Prescribing Information

Eylea 40 mg/ml solution for injection in a vial (aflibercept) Prescribing Information (Refer to full Summary of Product Characteristics (SmPC) before prescribing)

**Presentation:** 1 ml solution for injection contains 40 mg aflibercept. Each vial contains 100 microlitres, equivalent to 4 mg aflibercept. **Indication(s):** Treatment of neovascular (wet) age-related macular degeneration (AMD), macular oedema secondary to retinal vein occlusion (branch RVO or central RVO) and visual impairment due to diabetic macular oedema (DMO) in adults. **Posology & method of administration:** For intravitreal injection only. Must be administered according to medical standards and applicable guidelines by a qualified physician experienced in administering intravitreal injections. Each vial should only be used for the treatment of a single eye. The vial contains more than the recommended dose of 2 mg. The extractable volume of the vial (100 microlitres) is not to be used in total. The excess volume should be expelled before injecting. Refer to SmPC for full details. ***Adults***: The recommended dose is 2 mg aflibercept, equivalent to 50 microlitres. For wAMD treatment is initiated with one injection per month for three consecutive doses, followed by one injection every two months. No requirement for monitoring between injections. After the first 12 months of treatment, treatment interval may be extended based on visual and/or anatomic outcomes. In this case the schedule for monitoring may be more frequent than the schedule of injections. For RVO (branch RVO or central RVO), after the initial injection, treatment is given monthly at intervals not shorter than one month. Discontinue if visual and anatomic outcomes indicate that the patient is not benefiting from continued treatment. Treat monthly until maximum visual acuity and/or no signs of disease activity. Three or more consecutive, monthly injections may be needed. Treatment may then be continued with a treat and extend regimen with gradually increased treatment intervals to maintain stable visual and/or anatomic outcomes, however there are insufficient data to conclude on the length of these intervals. Shorten treatment intervals if visual and/or anatomic outcomes deteriorate. The monitoring and treatment schedule should be determined by the treating physician based on the individual patient's response. For DMO, initiate treatment with one injection/month for 5 consecutive doses, followed by one injection every two months. No requirement for monitoring between injections. After the first 12 months of treatment, the treatment interval may be extended based on visual and/or anatomic outcomes. The schedule for monitoring should be determined by the treating physician. If visual and anatomic outcomes indicate that the patient is not benefiting from continued treatment, treatment should be discontinued. ***Hepatic and/or renal impairment:*** No specific studies have been conducted. Available data do not suggest a need for a dose adjustment. ***Elderly population**:* No special considerations are needed. Limited experience in those with DMO over 75 years old. ***Paediatric population**:* No data available. **Contra-indications:** Hypersensitivity to active substance or any excipient; active or suspected ocular or periocular infection; active severe intraocular inflammation. **Warnings & precautions:** As with other intravitreal therapies endophthalmitis has been reported. Aseptic injection technique essential. Patients should be monitored during the week following the injection to permit early treatment if an infection occurs. Patients must report any symptoms of endophthalmitis without delay. Increases in intraocular pressure have been seen within 60 min of intravitreal injection; special precaution is needed in patients with poorly controlled glaucoma (do not inject while the intraocular pressure is≥30 mm Hg). Immediately after injection, monitor intraocular pressure and perfusion of optic nerve head and manage appropriately. There is a potential for immunogenicity as with other therapeutic proteins; patients should report any signs or symptoms of intraocular inflammation e.g pain, photophobia or redness, which may be a clinical sign of hypersensitivity. Systemic adverse events including non-ocular haemorrhages and arterial thromboembolic events have been reported following intravitreal injection of VEGF inhibitors. Safety and efficacy of concurrent use in both eyes have not been systemically studied. No data is available on concomitant use of Eylea with other anti-VEGF medicinal products (systemic or ocular). Caution in patients with risk factors for development of retinal pigment epithelial tears including large and/or high pigment epithelial retinal detachment. Withhold treatment in patients with: rhegmatogenous retinal detachment or stage 3 or 4 macular holes; with retinal break and do not resume treatment until the break is adequately repaired. Withhold treatment and do not resume before next scheduled treatment if there is: decrease in best-corrected visual acuity of ≥30 letters compared with the last assessment; central foveal subretinal haemorrhage, or haemorrhage ≥50%, of total lesion area. Do not treat in the 28 days prior to or following performed or planned intraocular surgery. Eylea should not be used in pregnancy unless the potential benefit outweighs the potential risk to the foetus. Women of childbearing potential have to use effective contraception during treatment and for at least 3 months after the last intravitreal injection. Populations with limited data: There is limited experience of treatment with Eylea in patients with ischaemic, chronic RVO. In patients presenting with clinical signs of irreversible ischaemic visual function loss, aflibercept treatment is not recommended. There is limited experience in DMO due to type I diabetes or in diabetic patients with an HbA1c over 12% or with proliferative diabetic retinopathy. Eylea has not been studied in patients with active systemic infections, concurrent eye conditions such as retinal detachment or macular hole, or in diabetic patients with uncontrolled hypertension. This lack of information should be considered when treating such patients. **Interactions:** No available data. **Fertility, pregnancy & lactation:** Not recommended during pregnancy unless potential benefit outweighs potential risk to the foetus. No data available in pregnant women. Studies in animals have shown embryo-foetal toxicity. Women of childbearing potential have to use effective contraception during treatment and for at least 3 months after the last injection. Not recommended during breastfeeding. Excretion in human milk: unknown. Male and female fertility impairment seen in animal studies with high systemic exposure not expected after ocular administration with very low systemic exposure. **Effects on ability to drive and use machines:** Possible temporary visual disturbances. Patients should not drive or use machines if vision inadequate. **Undesirable effects:** *Very common*: conjunctival haemorrhage (phase III studies: increased incidence in patients receiving anti-thrombotic agents), visual acuity reduced. *Common:* retinal pigment epithelial tear, detachment of the retinal pigment epithelium, retinal degeneration, vitreous haemorrhage, cataract (nuclear or subcapsular), corneal abrasion or erosion, corneal oedema, increased intraocular pressure, blurred vision, vitreous floaters, vitreous detachment, injection site pain, eye pain, foreign body sensation in eyes, increased lacrimation, eyelid oedema, injection site haemorrhage, punctate keratitis, conjunctival or ocular hyperaemia. *Uncommon:* Injection site irritation, abnormal sensation in eye, eyelid irritation. *Serious: cf. CI/W&P—in addition:* blindness, endophthalmitis, cataract traumatic, transient increased intraocular pressure, vitreous detachment, retinal detachment or tear, hypersensitivity (incl. allergic reactions), vitreous haemorrhage, cortical cataract, lenticular opacities, corneal epithelium defect/erosion, vitritis, uveitis, iritis, iridocyclitis, anterior chamber flare. Consult the SmPC in relation to other side effects. **Overdose:** Monitor intraocular pressure and treat if required**. Incompatibilities:** Do not mix with other medicinal products. **Special Precautions for Storage:** Store in a refrigerator (2 to 8 °C). Do not freeze. Unopened vials may be kept at room temperature (below 25 °C) for up to 24 h before use. **Legal Category:** POM. **Package Quantities & Basic NHS Costs:** Single vial pack £816.00. **MA Number(s):** EU/1/12/797/002. **Further information available from:** Bayer plc, Bayer House, Strawberry Hill, Newbury, Berkshire RG14 1JA, United Kingdom. Telephone: 01635 563000. **Date of preparation: March 2015**.Eylea is a trademark of the Bayer Group.Adverse events should be reported. Reporting forms and information can be found at www.mhra.gov.uk/yellowcard. Adverse events should also be reported to Bayer plc. Tel.: 01635 563500, Fax.: 01635 563703, E-mail: pvuk@bayer.com
